# Semiconductor Performance Optimization on Quasi-Two-Dimensional Bi_2_O_2_(S_*x*_Se_1–*x*_) through Monotonous Alloying

**DOI:** 10.1021/acs.nanolett.5c01164

**Published:** 2025-05-09

**Authors:** Yong-Jyun Wang, Li-Lun Chu, Yu-Hao Tu, Li-Hui Tsao, Ming-Kuan Fan, Wei-Ting Chen, Chien-Wei Chen, Chan-Yuen Chang, Yuan-Chih Chang, Yu-Lun Chueh, Po-Wen Chiu, Chao-Hui Yeh, Ying-Hao Chu

**Affiliations:** † Department of Materials Science and Engineering, 34881 National Tsing Hua University, Hsinchu 300044, Taiwan; ‡ College of Semiconductor Research, 34881 National Tsing Hua University, Hsinchu 300044, Taiwan; § Taiwan Instrument Research Institute, 87786 National Applied Research Laboratories, Hsinchu 300092, Taiwan; ∥ Department of Electrical Engineering, 34881 National Tsing Hua University, Hsinchu 300044, Taiwan; ⊥ Institute of Electronics Engineering, 34881 National Tsing Hua University, Hsinchu 300044, Taiwan; # Department of Physics, National Sun Yat-Sen University, Kaohsiung 804201, Taiwan; 7 Department of Materials Science and Engineering, Korea University, Seoul 02841, Republic of Korea

**Keywords:** Bi_2_O_2_Se, Bi_2_O_2_S, 2D alloy, semiconductor device

## Abstract

Bismuth oxychalcogenides (Bi_2_O_2_X, where X = S, Se, Te) have garnered significant attention recently due to their high electron mobility, air stability, and excellent photoelectric properties. Therefore, precise control and optimization over the properties of these novel quasi-2D materials are crucial for practical applications. In this study, we synthesize epitaxial films of Bi_2_O_2_(S,Se) by the monotonous alloying of sulfur (S) and selenium (Se). Our findings reveal that the lattice constants, band gaps, and electrical properties of the films vary according to the elemental composition. Further, we observed an enhanced field-effect mobility of ∼215 cm^2^/(V s) and an on/off ratio of ∼10^6^ in the Bi_2_O_2_(S_0.4_Se_0.6_) heterostructures with a Bi_2_SeO_5_ (BSO) oxide layer. With these efforts, this work establishes a pathway toward developing novel designs for 2D Bi_2_O_2_X materials.

## Graphical abstract

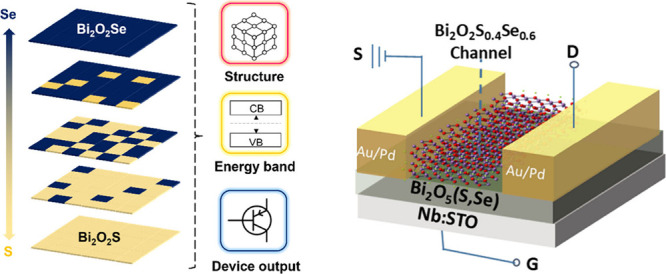

Due to the boost of AI applications, high-speed and efficient computation is in great demand. However, Si-based CMOS technology faces challenges, including performance degradation due to the shrinkage of the body thickness.^1,2^ Therefore, the adoption of new semiconductors is critical. Since the discovery of graphene, the field of two-dimensional (2D) materials has emerged prominently and captivated researchers.^3–5^ The search for 2D semiconductors with excellent electrical performance and stability in ambient environments is urgent. Bi_2_O_2_Se (BOSe), an air-stable layered oxide, stands out as a promising semiconductor due to its exceptional electronic properties.^6,7^ Its inherent layered structure is conducive for fabricating electronic devices, even at the scale of just a few atomic layers.^8–10^ Notably, the BOSe-based top-gated field-effect transistor (FET) boasts remarkable properties, such as high carrier mobility (reaching ∼28,900 cm^2^/V·s at 1.9 K and 450 cm^2^/V·s at room temperature) and an impressive current on/off ratio of >10^6^, with an almost ideal subthreshold swing (SS) (∼65 mV/dec).^6^ Furthermore, the moderate bandgap (∼0.8 eV) of BOSe ensures its suitability for room temperature operations while maintaining a relatively low operational voltage.^6,11^ Given its intriguing properties, chemical stability in ambient conditions, and ease of accessibility, BOSe emerges as a rising star for the next generation of compact, high-performance, and energy-efficient electronic devices.^12–21^ Notably, Bi can be substituted or mixed with elements such as La and Sb, while the Se atom can be replaced with S or Te,^22–24^ implying a path to expand the design of its functionalities. Thus, in this study, a monotonic alloying approach is adopted to optimize the semiconductor characteristics of Bi_2_O_2_(S_*x*_Se_1–*x*_) (BO­(S,Se)). Our findings reveal that the lattice constant, band gap, and electrical properties of thin films show a relation with composition concentrations. Further, there is a noticeable increase in field-effect mobility to 215 cm^2^/V·s while the S/Se ratio is 4:6. Meanwhile, the native oxide layer Bi_2_SeO_5_ can also be secured, showing superior dielectric properties and excellent compatibility with the BO­(S,Se). With these efforts, a new idea for modulating electronic properties is orchestrated, invoking further development of leading-edge semiconductor-on-insulator 2D FETs and inspiring future research in this field.

A commercial SrTiO_3_ (STO) substrate is adopted for epitaxial growth due to its excellent lattice compatibility with BOSe and Bi_2_O_2_S (BOS) to promote the heterointerface quality during film stacking for subsequent measurements. A dual-target pulsed laser deposition was introduced to control the composition of BO­(S,Se) solid solutions, as shown in Figure 1(a).^25,26^ The composition can be designed by varying the laser pulses of each target. The sample homogeneity is achieved through interdiffusion between the Se and S species promoted by substrate heating during the deposition. The epitaxial characteristics of the BO­(S,Se)/STO heterostructure were investigated by X-ray diffraction (XRD). The θ–2θ scan shown in Figure 1b indicates that only the BO­(S,Se) (00L) series signals appear without other secondary phases besides the STO (00L) signals, delivering the orientation relationship of BO­(S,Se)(001)||STO(001). The pristine BO­(S,Se) phases are located in the ranges of BOS (00L) and BOSe (00L). By applying Bragg’s law, we can convert the results of the θ–2θ scan into lattice constants. Typically, the lattice change with composition in monotonous alloying can be described by Vegard’s law. However, we observed a significant positive deviation, as shown in Figure 1c, suggesting a lattice expansion along the out-of-plane direction. The positive deviation appears when the ***a*** and ***b*** axes are relatively severely changed, especially in Table S1. Though the bonding nature of the BO­(S,Se) film and STO substrate is not similar to the conventional covalent bonds in oxide epitaxy, the growth of BOSe should be slightly influenced by the substrate, further leading to the change of ***a*** and ***b*** axes, respectively. Furthermore, the heterostructure performance strongly depends on the crystal quality. Thus, the rocking curve measurement of the BO­(S,Se)/STO heterostructures was conducted, and the results are shown in Figure 1d. The full width at half-maximum (fwhm) of BO­(S,Se) (006) peaks is ∼0.1° for the whole composition range, indicating the superior crystallinity of the heterostructure. The phi scans were used to investigate the epitaxial feature, and the result is shown in Figure S1. The reflection of BO­(S,Se){110} can be detected every 90°, indicating a single structure domain, since the (001)-oriented BO­(S,Se) film shows a 4-fold symmetry. The perfect alignment between STO {110} and BO­(S,Se) {220} peaks at every 90° interval confirms the in-plane epitaxial relationship as BO­(S,Se) [010]||STO[010], providing the critical evidence of heteroepitaxy. Reciprocal space mappings (RSMs) further investigated the substrate constraint, as shown in Figure 1e and Figure S1. The superior lattice match of STO and BOSe caused perfect alignment of the in-plane direction. In contrast, the pristine BOS shows misalignment of the in-plane direction, suggesting a strain relaxation. However, the alignment of BO­(S_0.5_Se_0.5_) and STO in the in-plane direction suggests a dominant substrate effect, the primary reason for the positive deviation from Vagard’s law. Based on a series of XRD tests, evidence of superior heteroepitaxy with the epitaxial relationship of BO­(S,Se)[010]||STO[010] and BO­(S,Se) [001]||STO[001] is delivered, as shown in Figure 1f.

**Figure 1:**
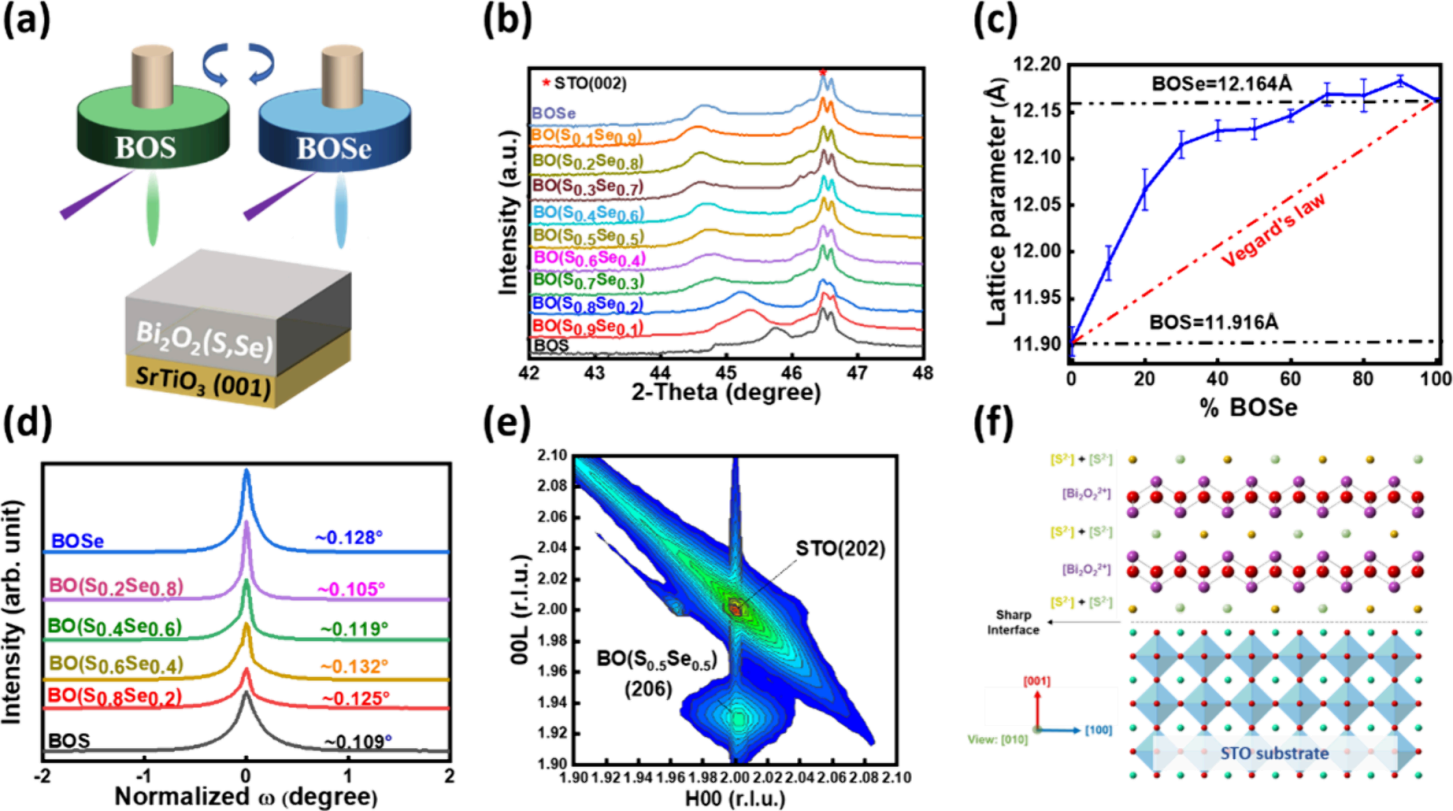
(a) Schematic diagram of the BO­(S,Se) fabrication. (b) XRD θ–2θ scans, (c) lattice parameters, (d) rocking curves, and (e) RSM mapping of the BO­(S,Se)/STO heterostructures. (f) The schematic diagram of the BO­(S,Se)/STO heterostructure with an epitaxial relationship.

Furthermore, the interface microstructure of the heterostructure was investigated by transmission electron microscopy (TEM). Figure 2a shows the cross-sectional TEM image of the synthesized heterostructure and the corresponding Fourier transform (FFT) diffraction patterns. The sharp interface between the BO­(S,Se) film and substrate can be observed. The BO­(S,Se) energy-dispersive spectroscopy (EDS) mapping (Figure S2) suggests a uniform distribution of S and Se without noticeable interdiffusion to the substrate. Moreover, the reciprocal lattices in the FFT patterns of the BO­(S,Se) and STO layers are indexed in the insets, indicating the epitaxial relationship and delivering consistent results with XRD. These efforts have established the correctness of phases and confirmed the epitaxial relationships. After that, X-ray photoelectron spectroscopy (XPS) was conducted to determine the chemical state of the BO­(S,Se) films (Figure 2b–e). The valence states of Bi, Se, and S are +3, −2, and −2, respectively, consistent with the previous report.^27,28^ The chemical environment of the synthesized solid solution is different compared with the pristine one (Figure S3). A shifted peak toward a negative direction can be observed in Bi, O, and Se. This can be attributed to the fact that the insertion of S into BOSe enlarges the lattice, which might lower the binding energy of each atom. With these efforts, the influence of S on the electric potential can be investigated and discussed.

**Figure 2:**
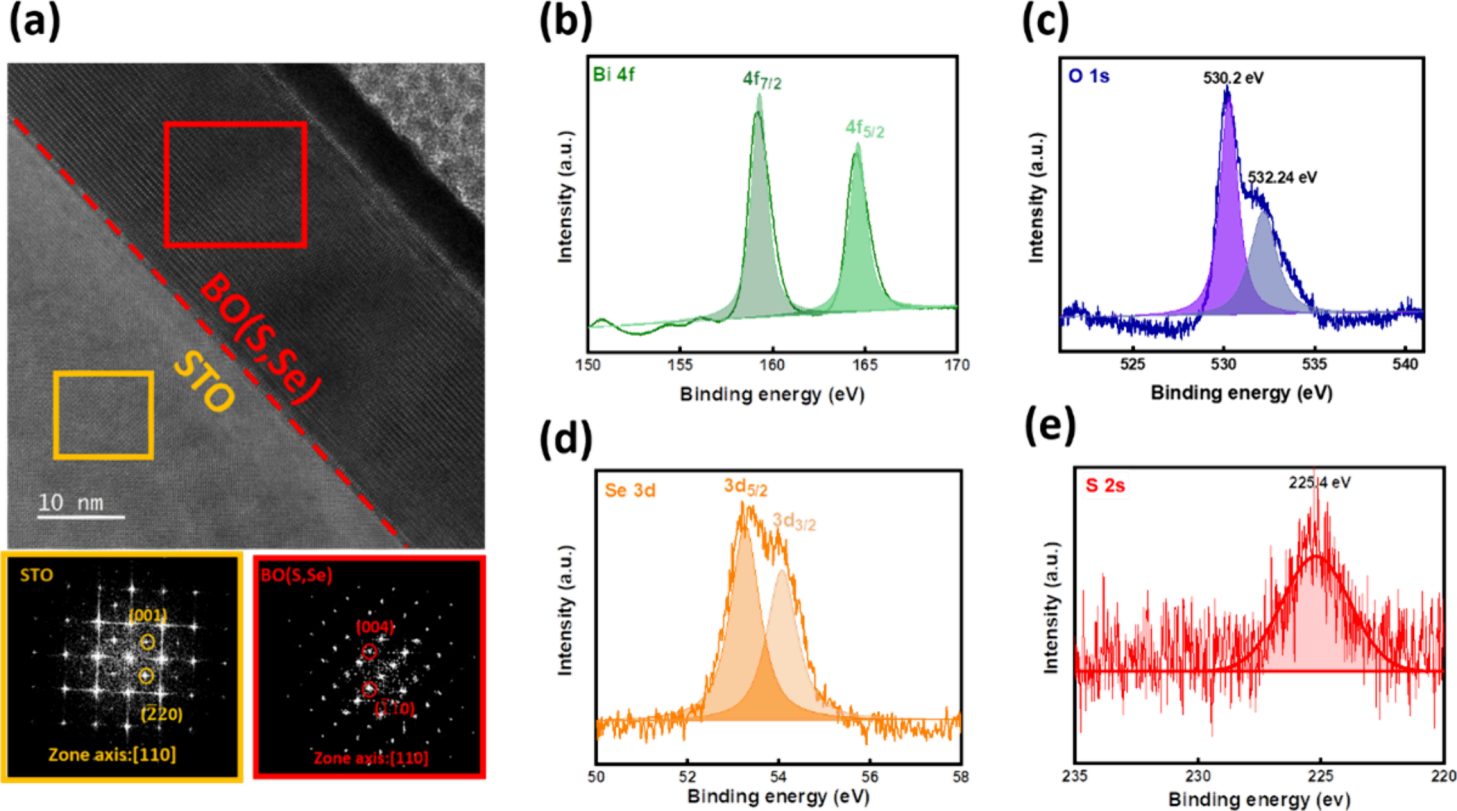
(a) Cross-sectional TEM image and the corresponding FFT patterns of the BO­(S,Se)/STO heterostructure. (b–e) XPS spectra of the BO­(S,Se) film.

After probing the structural change as a function of composition, we aim to investigate the electronic structure with composition tuning. The optical bandgap of the BO­(S,Se) system was studied using UV–vis spectroscopy combined with the Tauc plot analysis. It can be expressed using the Kubelka–Munk function as

(αhν)1/n=A(hν−Eg)

Here, α is the absorption coefficient; *h*ν is the photon energy; Eg is the optical bandgap; *A* is a constant; and *n* is a parameter determined by the electronic transition type. Six distinct compositions were selected to investigate the compositional effect on the optical properties, as shown in Figure S4. Figure 3a shows the bandgap variation of BO­(S,Se) along with the composition. The pristine BOS and BOSe optical bandgaps are ∼1.6 and ∼1.1, respectively, consistent with the reported values.^29,30^ The results reveal a progressive increase in the optical bandgap as the composition transitions from BOSe to BOS. According to Vegard’s law, this trend is accompanied by an observable positive deviation in the overall bandgap behavior, indicating that the Se-to-S ratio within the system strongly influences the optical properties due to the crystal structure modification (see Supporting Information Figure S5). These findings provide valuable insight into the tunability of the bandgap in BO­(S,Se) compounds, which is critical for potential applications in optoelectronic devices. Since the change in bandgap suggests the tuning of electrical properties, a comprehensive series of electrical measurements was conducted to evaluate the transport properties of the BO­(S,Se) system. Based on the Hall measurement results (Figure 3b, 3c), we found an increase in sheet resistance with S addition, as shown in Figure S6, and the corresponding Hall mobility of BO­(S,Se) films was extracted along with the composition change. For the film thickness of 60 nm, BOSe exhibits an electron mobility of ∼145 cm^2^/V·s, which aligns closely with reported values in the literature.^31^ Interestingly, when the Se:S ratio reaches ∼6:4, the electron mobility achieves its maximum value of ∼198 cm^2^/V·s, highlighting the critical role of composition in optimizing the transport properties. To push device fabrication, channel mobility is more crucial. Thus, we fabricated a top-gate field-effect transistor (FET) structure using 8 nm BO­(S,Se) with a 15 nm HfO_2_ dielectric layer to validate this observation further. As shown in Figure 3f, the field-effect mobility extracted from our devices exhibits a composition-dependent trend similar to that observed in the Hall measurements, with the highest field-effect mobility occurring at an S/Se ratio of 4:6. (The details of the mechanism are shown in Supporting Information Figures S7 and S8.) Figures 3d and 3e present the transfer characteristics of BOS_0_._4_Se_0_._6_, demonstrating a high field-effect mobility of 252 cm^2^/V·s along with an impressive on/off ratio of 7 orders of magnitude. However, it is also observed that the SS value reaches as high as 842 mV/dec (the interface defect density is calculated in Supporting Information Figure S9). Therefore, we aim to optimize the dielectric layer to further reduce the SS value.

**Figure 3:**
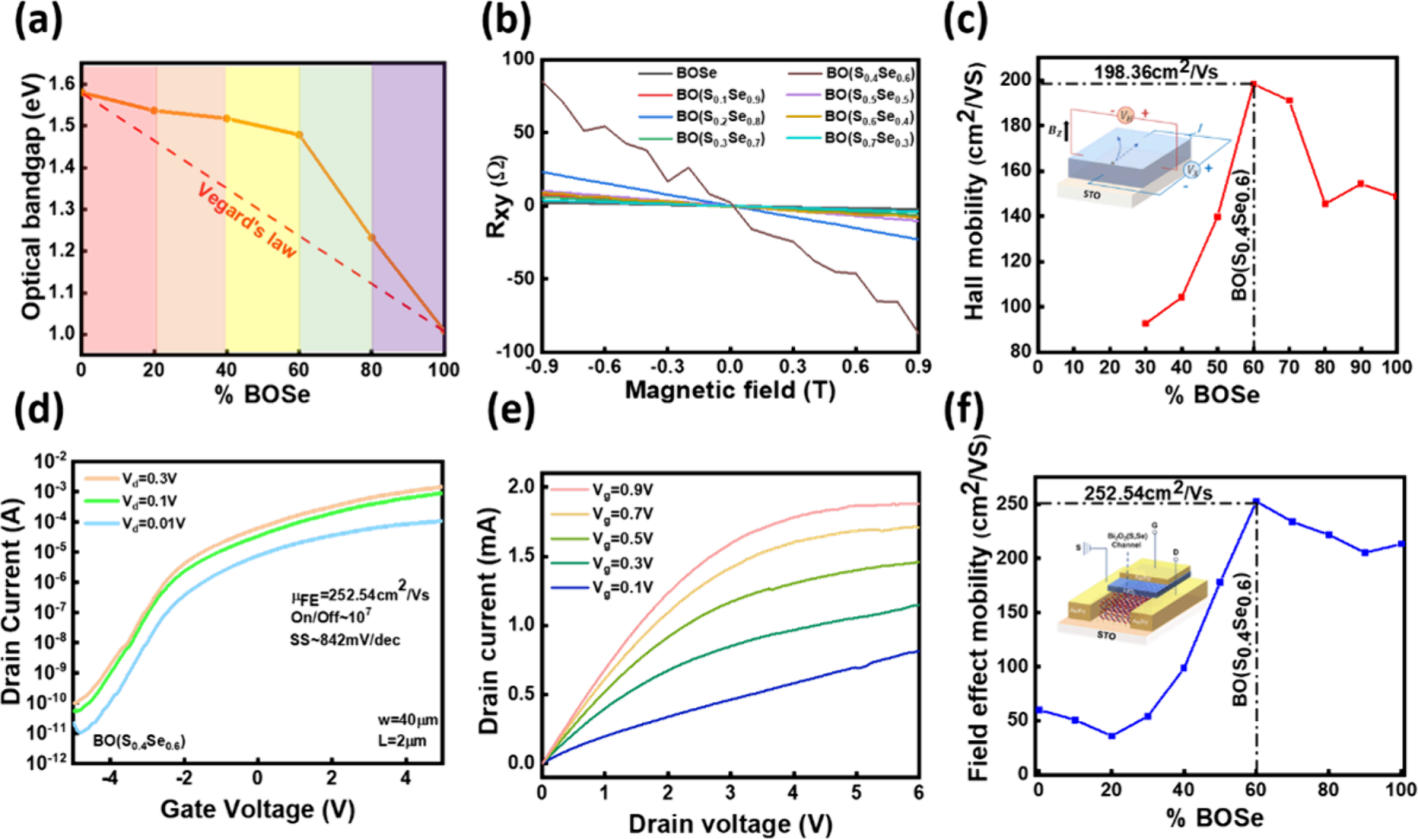
(a) Bandgap of BO­(S,Se) along with different compositions. (b) The Hall measurement results of the BO­(S,Se)/STO heterostructures. (c) The corresponding Hall mobility of BO­(S,Se) with different compositions. (d,e) The transfer and output characteristics of the HfO_2_/BO­(S_0.4_Se_0.6_)/STO top-gate FET. (f) Field-effect mobility of BO­(S,Se) extracted from the top-gate FET devices.

After realizing the influence of composition on the electronic structure and transport properties, we identified the Se:S ratio of 6:4 as the optimal composition. We utilized this composition for subsequent device fabrication. Credited to the high electron mobility (198 cm^2^/V·s), a FET device with a bottom-gated device structure can be demonstrated, as shown in Figure 4a. The high-*k* dielectric BSO layer is adopted due to the compatible lattice and sharp interface with BO­(S,Se), and the structure verification and dielectric response are shown in Figure S10. The formation of this series of native oxides is a key advantage of Bi_2_O_2_X semiconductors.^8,32,33^ Meanwhile, for the metal contact, Au (work function ∼ 5.1 eV)/Pd (work function ∼ 5.2 eV) is used for the source and drain electrodes to form ohmic contacts. The thickness of Pd is ∼15 nm, and it serves as the first contact due to its closest work function to BO­(S,Se). After that, the Au was deposited for ∼20 nm, a metal with air stability. The transfer curves of the BO­(S,Se)-based N-FET with the optimized thickness of BO­(S,Se) for ∼8 nm are shown in Figure 4b. The transfer curves of the N-FET show typical n-type behaviors with field-effect electron mobility for ∼215 cm^2^/V·s, an on/off ratio of 1E6, and an SS value of ∼ 100 mV/dec Also, the linear and saturation regions can be observed in *I*_ds_–*V*_ds_ (Figure 4c) along with increasing gate voltages, suggesting superior gate control by the native BSO to the pristine n-type BO­(S,Se) channel. The comparison of the device characteristics (*I*_on_/*I*_off_ and field-effect mobility) is shown in the benchmark in Figure 4d.^6,34–43^ The electron mobility in the BO­(S,Se)-based N-FET device is higher than that in most N-FETs based on 2D materials while maintaining a moderate *I*_on_/*I*_off_ ratio.

**Figure 4:**
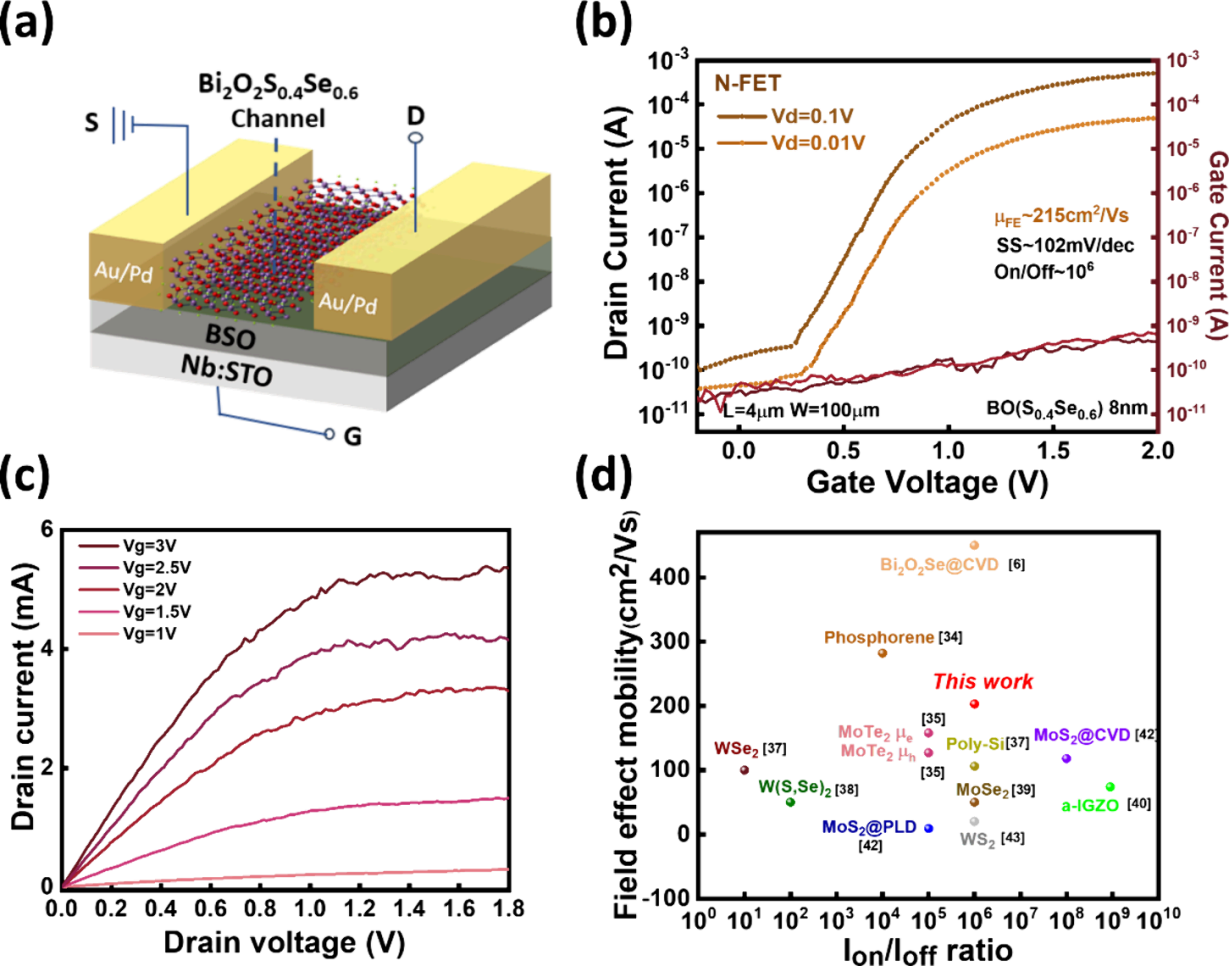
Transfer characteristics of the bottom gate BO­(S,Se)/BSO/Nb:STO transistor device. (a) The schematic diagram of the bottom-gated device structure. (b,c) The transfer and output characteristics of the synthesized N-FET. (d) Benchmark of the field-effect mobility versus on/off ratio for the N-FETs.

This work reports the BO­(S,Se) solid solution with compositional variation for tuning the electronic behaviors. After a series of structural and compositional characteristics are identified, the epitaxial relation and precise compositional modulation can be identified. The modulation of the bandgap and transport properties of the BO­(S,Se) solid solution depend on the compositional change. Based on the results of transport measurements, an optimized composition with S:Se = 4:6 can be determined, showing the highest electron mobility (198 cm^2^/V·s) compared to other compositions. Thus, a transistor device with S:Se = 4:6 has been demonstrated. The transfer characteristics of the synthesized N-FET show superior field-effect mobility (215 cm^2^/V·s) with an on/off ratio of ∼6 orders. With these efforts, a pathway to optimize electronic behaviors through composition modulation is expected to push the next-generation electronics.

## Sample Preparation

The epitaxial BO­(S,Se) solid solutions were fabricated via PLD dual-target deposition with commercial BOSe and BOS targets. Commercial STO single crystals were used as the substrates. The STO and 0.5 wt % Nb-doped STO substrates with 99.99% purity were secured from Eternal Stars International Co., Ltd. On the other hand, the targets with 99.99% purity used in this work are made by Ultimate Materials Technology Co., Ltd. The cleaned substrate was immediately loaded into the processing chamber to minimize contamination. A KrF excimer laser (λ = 248 nm; Lambda Physik, Coherent agent) was operated at a 10-Hz repetition rate and energy density of 1 J/cm^2^. The vacuum chamber was evacuated to a pressure of 1 × 10^–6^ Torr before deposition. The BOSe was grown on an STO substrate at 405 °C under 100 mTorr of O_2_ pressure. After the deposition of BOSe, the temperature was maintained at the original 415 °C, and the oxygen pressure of the chamber was increased from 100 mTorr to 100 Torr, employing this thermal oxidation for about 3 h, and then cooled down at 0.1 °C/s. Such a process ensures the complete oxidation of BOSe into the BSO dielectric layer. The BO­(S,Se) alloy was grown on an STO substrate at 415 °C under 50 mTorr of O_2_ pressure. Lastly, the cooling process was conducted with a cooling rate of 0.3 °C/s.

## Fabrication of the HfO_2_ Layer

A 2 in. atomic layer deposition (ALD) system, developed in-house by the Taiwan Instrument Research Institute, was used for the deposition process. Tetrakis­(ethylmethylamido) hafnium (TEMAHf) served as the hafnium precursor, sourced from PentaPro Materials, with deionized water (H_2_O) as the oxidant. The TEMAHf precursor has a boiling point of 78 °C at 0.01 hPa, and to prevent precursor condensation within the delivery lines, the precursor cylinder, gas lines, and chamber lid were maintained at 120 °C. The substrate was heated to 200 °C before initiating the deposition process. The ALD growth rate was determined to be 0.9 Å/cycle, achieving a 10 nm film thickness after 110 cycles. Film thickness on the Si substrate was monitored by using a SENTECH SENresearch 4.0 ellipsometer.

## X-ray Diffraction in NSRRC

The crystal structure and phase identification were characterized by synchrotron-based X-ray diffraction techniques at beamlines 13A and 17A in the National Synchrotron Radiation Research Center in Hsinchu, Taiwan. The incident beam was monochromated at 9.3 keV (ca. 1.333 Å) with a Si(111) double-crystal mirror and then focused by a toroidal focusing mirror to obtain a higher-intensity beam. Four sets of slits were used to gain the detection resolution, where two sets of slits were placed in front of the samples to set beam size and the other two placed after the sample (or before the scintillation counter) to decrease background noise. These diffraction measurements were plotted in reciprocal lattice units normalized to the STO substrate (1 r.l.u. = 2π/a STO).

## X-ray Photoelectron Spectroscopy (XPS)

After the load lock was evacuated to 3 × 10^–1^ Torr, the sample was transferred to the transfer chamber under vacuum conditions. The pressure was further reduced to 3 × 10^–6^ Torr before the sample was transferred into the XPS analysis chamber. Once the pressure dropped below 1 × 10^–7^ Torr, elemental analysis of the thin film was performed using a SPECS XPS system (PHOIBOS 150 WAL 2D-CMOS). The system setup included: Flood Gun Power Supply, COSCON FG; X-ray Source, Mg target (SPECS XR 50, spot size ∼ 10 mm × 10 mm); Analyzer, Omicron EA125; Analyzer Lens Mode, High-Intensity Mode (3.5 kV); Pass Energy, 60 eV; Survey Scan Step Size, 0.5 eV; Narrow Scan Step Size, 0.02 eV; Dwell Time, 96 ms.

## Transmission Electron Microscopy

The TEM sample was prepared by using the FEI Helios 600i Dual Beam system. A platinum protective layer was deposited on the top surface of the sample by using a gas injection system (GIS). A 30 keV gallium ion beam was used for rough milling, and a 5 keV ion beam was used for polishing. The sample was then characterized by a JEOL JEM-F200 transmission electron microscope. The zone axis was calibrated by using a double-tilt sample stage. The bright-field high-resolution TEM image was obtained under 200 keV. For energy-dispersive spectroscopy (EDS), an Oxford X-Max 80 mm^2^ EDS detector was used to collect the signal under scanning transmission electron microscope mode.

## Device Fabrication

After sample preparation, the sample was exposed by using a Digital Light Processing Maskless Exposure System. Subsequently, the etching process was performed using a buffered oxide etching technique for 20 s. Next, electrode deposition was carried out in an evaporation system under a pressure lower than 5 × 10^–6^ Torr. Fifteen nm of Pd was deposited at a rate of 0.2 Å/s, followed by 20 nm of Au at a rate of 0.3 Å/s. Finally, the photoresist was removed by acetone and IPA. The detailed process flow for the top-gate and bottom-gate FET can be found in Figure S11.

## Electrical Property

The sheet resistance and Hall measurements were performed using a Quantum Design PPMS. The sample was mounted on the standard PPMS puck. The longitudinal resistance was measured using a four-probe method, and the Hall measurement was conducted by using the classic Hall-bar geometry. To obtain the performance of the synthesized FET device, the transfer characteristics were measured using a Keysight B1500A semiconductor analyzer.

## Supplementary Material



## Data Availability

All the data needed to evaluate the conclusions in the paper are present in the paper and the Supporting Information.

## Notes

Details of structural information include phi scans, RSM, and cross-sectional EDS images are shown in Figures S1 and S2. Figure S3 reveals the valence states of BO­(S, Se) through XPS spectra. The Tauc plot results for extracting bandgap are shown in Figure S4, along with the explanation of positive deviation in the band gap shown in Figure S5. Figure S6 presents the sheet resistance of each composition. The simulated result of electronic structure and electron mobility of BO­(S, Se) are shown in Figures S7 and S8. Figure S9 shows the estimation of the interfacial defect density between HfO_2_/BO­(S,Se). The structural characterization and dielectric properties of the BSO/STO heterostructure are shown in Figure S10. Figure S11 illustrates the device fabrication process flow (PDF)
