# Supplementary material for: Semiconductor Performance Optimization on Quasi-Two-Dimensional Bi2O2(S x Se1–x ) through Monotonous Alloying
Source: Nano Lett. 2025 May 9;25(20):8186–93. doi: 10.1021/acs.nanolett.5c01164 (PMC12100717; doi:10.1021/acs.nanolett.5c01164)
Supplement: Supplementary file 1 [file nl5c01164_si_001.pdf]

Semiconductor Performance Optimization on Quasi-Two-Dimensional $Bi_2O_2(S_xSe_{1-x})$ through Monotonous Alloying

Yong-Jyun Wang[1], Li-Lun Chu[1], Yu-Hao Tu[1], Li-Hui Tsao[1], Ming-Kuan Fan[1], Wei-Ting Chen[2], Chien-Wei Chen[3], Chan-Yuen Chang[3], Yuan-Chih Chang[4,5], Yu-Lun Chueh[1,6,7], Po-Wen Chiu[4], Chao-Hui Yeh[2,4,5*], Ying-Hao Chu[1,2*]

[1]Department of Materials Science and Engineering, National Tsing Hua University, Hsinchu 300044, Taiwan

[2]College of Semiconductor Research, National Tsing Hua University, Hsinchu 300044, Taiwan

[3]Taiwan Instrument Research Institute, National Applied Research Laboratories, Hsinchu, 300092, Taiwan

[4]Department of Electrical Engineering, National Tsing Hua University, Hsinchu 300044, Taiwan

[5]Institute of Electronics Engineering, National Tsing Hua University, Hsinchu 300044, Taiwan

[6]Department of Physics, National Sun Yat-Sen University, Kaohsiung, 804201, Taiwan.

[7]Department of Materials Science and Engineering, Korea University, Seoul 02841, Republic of Korea.

*Correspondence to: yhchu@mx.nthu.edu.tw and chyeh@ee.nthu.edu.tw

Structural information

The phi scans shown in Figure S1a were used to verify the epitaxial feature further to determine the in-plane (IP) orientation. The four-fold symmetry along (001) orientation is observed, and four sets of peaks at 90° intervals are displayed. Figures S1b and S1c are the reciprocal space mappings (RSMs) of the BOSe/STO heterostructure and the BOS/STO heterostructure, respectively.

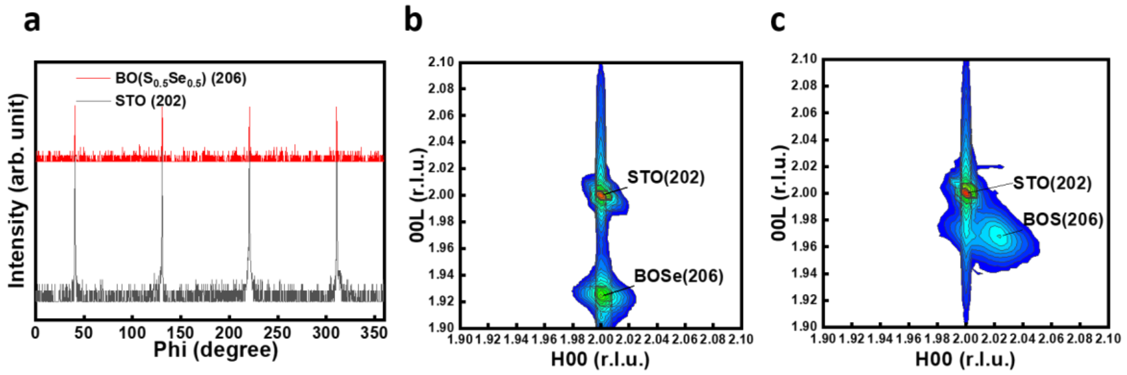

Figure S1. a. phi scans of the BO(S,Se)/STO sample. b. RSM mapping of the BOSe/STO heterostructure. c. RSM mapping of the BOS/STO heterostructure.

Table S1. The lattice constants of BO(S,Se) for each composition.

S(%)	a	b	c
0	3.905	3.881	12.162
0.125	3.89	3.857	12.17
0.25	3.89	3.857	12.162
0.375	3.8825	3.845	12.145
0.5	3.885	3.833	12.132
0.625	3.87	3.835	12.1
0.75	3.872	3.845	12.018
0.875	3.872	3.855	11.957
1	3.859	3.864	11.904

Cross-sectional EDS images of BO(S,Se)/STO sample

We conducted a cross-sectional TEM-EDS analysis to further investigate the distribution of S and Se in the BO(S,Se) thin film and obtain elemental mapping and

structural information. As shown in Figure S2a, the cross-sectional structure of the Pt/BO(S,Se)/STO sample reveals a well-defined and smooth BOSe/STO interface. The EDS mapping results, presented in Figure S2b–g, demonstrate that the spatial distribution of S and Se is not in a layer-by-layer manner but relatively homogeneous throughout the film. Furthermore, the sum of the concentrations of S and Se is approximately half that of Bi, which is consistent with the expected stoichiometry of the BO(S,Se) thin film.

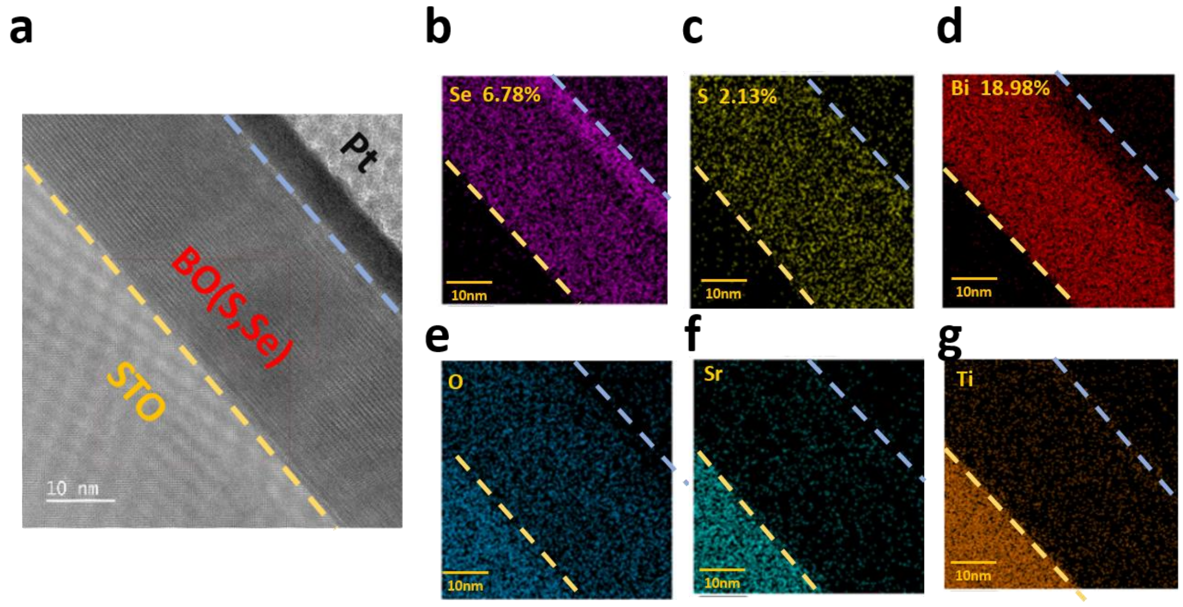

Figure S2. a. Cross-sectional image of the BO(S,Se)/STO sample. b-g. Cross-sectional TEM EDS mapping of the BO(S,Se)/STO sample.

XPS for pristine BOSe

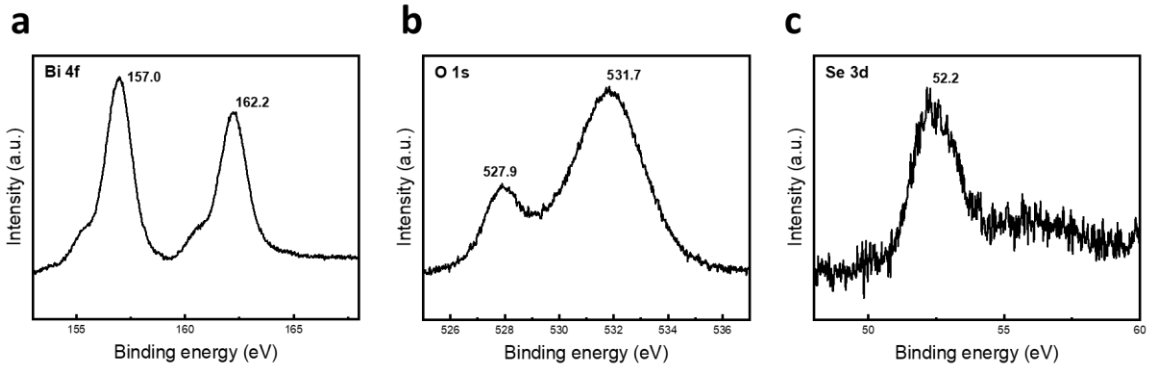

Figure S3. XPS spectra of the pristine BOSe.

The Tauc plot result

The optical bandgap was determined using UV-Vis absorption spectroscopy, where the absorbance as a function of wavelength was measured and subsequently analyzed using the Tauc plot method. Figure S4a–f present the Tauc plot results, with the exponent n set to 1/2 in the Kubelka–Munk function to calculate the direct bandgap energy.

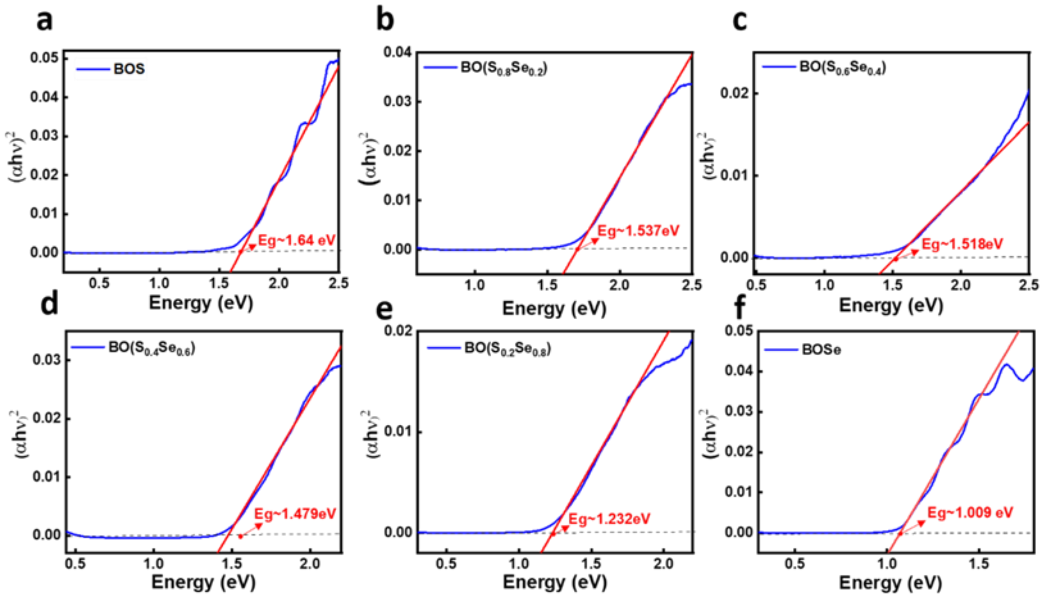

Figure S4. The Tauc plot result of a. BOS, b. BO($S_{0.8}Se_{0.2}$), c. BO($S_{0.6}Se_{0.4}$), d. BO($S_{0.4}Se_{0.6}$), e. BO($S_{0.2}Se_{0.8}$), f. BOSe.

The explanation of positive deviation in the band gap

The S/Se ratio has been modulated in the lattice. It is noted we found that the band gap strongly depends on reduced ***a*** and ***b*** axes, functions of the S/Se ratio and the S and Se arrangements. If the arrangement of S and Se has been arranged at a fixed composition, the positive deviation would appear, similar to the behavior shown in the original manuscript, as shown in Figure S5.

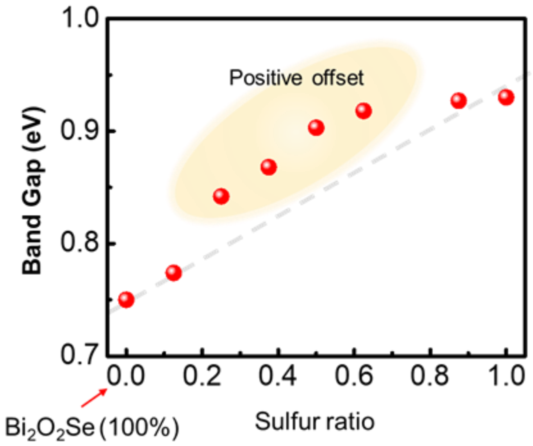

Figure S5. The simulation results of $BO(S_xSe_{1-x})$'s band gap.

The sheet resistance of each composition

Figure S6 presents the variation of sheet resistance as a function of composition. The overall sheet resistance sharply decreases with a slight addition of Se to BOS. As the Se concentration increases, the decreasing trend gradually stabilizes. This result further confirms that the electrical properties of the sample can be effectively tuned by adjusting the S/Se ratio.

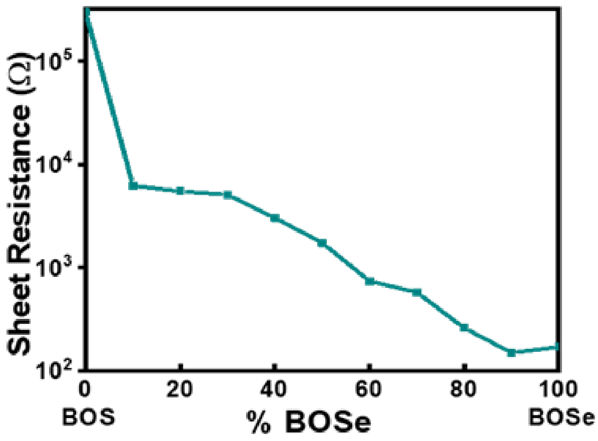

Figure S6. The sheet resistance of the BO(S,Se) films with different compositions.

The simulated result of mobility for each composition

The introduction of S into the BOSe lattice would lead to the re-hybridization of the electron orbital, subsequently adjusting the band structure. In this way, the effective mass of the electron would be changed based on the amount of S, which is directly influenced by the curvature of the band structure. On the other hand, in terms of the stress effect of epitaxial films on hetero-substrates, the lattice-mismatch-induced compressive strain in as-grown BO(Se,S) could be further intensified, particularly with a change of the ratio of Se and S atoms in this BO(Se, S) hybridization system. This strain effect can reconfigure electronic band structures, thereby altering the effective mass of carriers. Our relevant simulations have demonstrated modulated bandgap energies at the Gamma point (Γ), as well as changes in the curvatures of the energy bands (Figure S7), further influencing the effective mass (m^*) and electron transport. Additionally, we found that the local built-in electric field caused by the local disorder of S/Se would impact the electronic behaviors. Thus, we have considered all factors and conducted the simulation, as shown in Figure S8. The highest electron mobility would be observed in certain components.

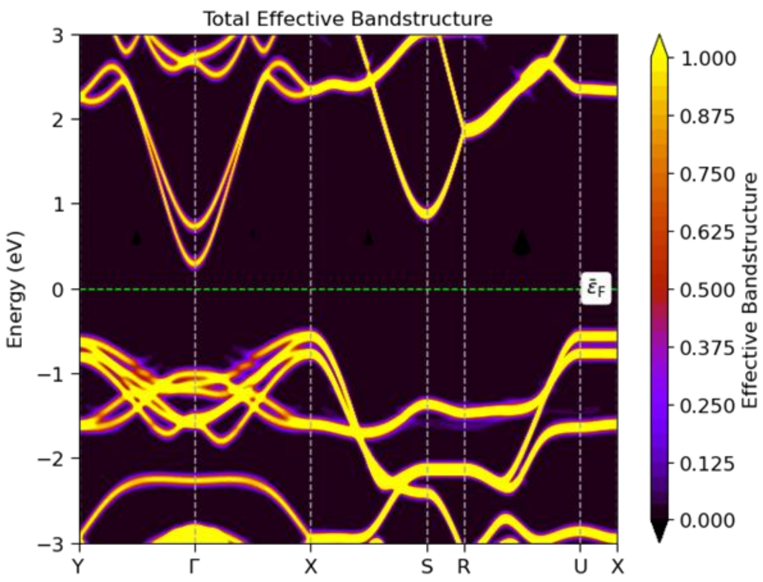

Figure S7. The simulated band structure of the synthesized BO(S,Se).

Leveraging the calculation outputs by Quantum ATK ($mobility \propto \frac{\tau}{m^*}$, where "τ" stands for relaxation time), our simulation results clearly show that the mobility distribution peaks at around 60-80% Se concentration in the BO(Se,S) alloy, as shown in Figure S8. This corresponds well with the experimental transport properties, specifically the carrier mobility extracted through the Hall measurements. Therefore, the highest mobility observed at an S/Se ratio of 4/6 can be attributed to the abovementioned factors.

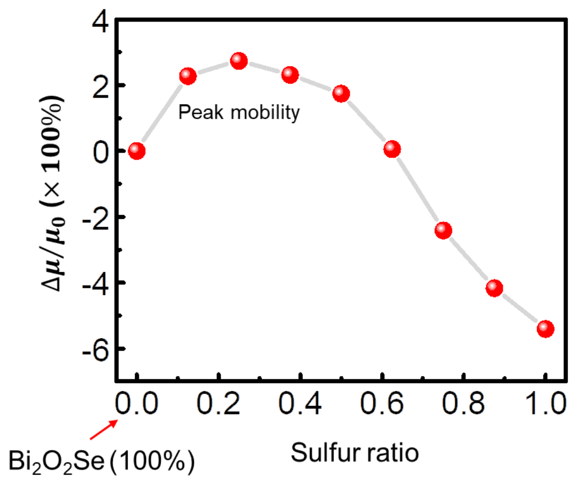

Figure S8. The simulation results of $BO(S_xSe_{1-x})$'s mobility.

Estimation of the interfacial defect density between HfO_2/BO(S,Se)

We calculated the defect density at the HfO_2/BO(S, Se) interface. The defect density can be evaluated through this formula:

$$D = \frac{C_{OX}}{q} \left(\frac{1}{C_{LF}} - \frac{1}{C_{HF}}\right)$$

where D is the defect density ($cm^{-2}eV^{-1}$), C_{OX} is the capacitance density of HfO_2, q is the Coulombic constant (C), C_{LF} and C_{HF} are the capacitance density of HfO_2/BO(S, Se) under low (0.1 kHz) and high frequency (1 MHz). Figure S9 are the capacitance measurement results of the HfO_2 and HfO_2/BO(S,Se) samples under low (0.1 kHz) and high frequency (1 MHz). Based on the results, the defect density is calculated as 4.8×10^{13} $cm^{-2}eV^{-1}$, suggesting that many defects exhibit at the interface of HfO_2/BO(S, Se). Thus, the output subthreshold swing (SS) value would be high.

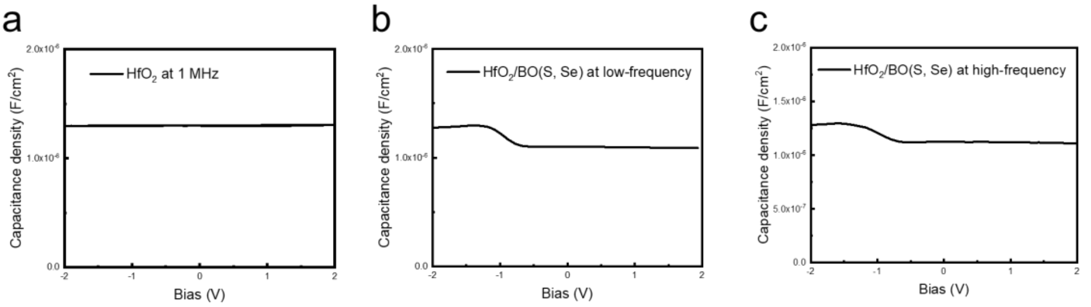

Figure S9. The capacitance measurement results of the HfO_2 and HfO_2/BO(S,Se) samples under low (0.1 kHz) and high frequency (1 MHz).

The structural characterization and dielectric properties of the BSO/STO heterostructure

Before device fabrication, the dielectric layer BSO properties were characterized. Figure S10a presents the θ-2θ scan results of the BSO/STO heterostructure, revealing

that BSO grows along the STO (00L) direction without any observable secondary phases or impurities. Figure S10b shows the Capacitance-Voltage (C-V) measurement of the BSO thin film, where the capacitance remains highly stable under an applied voltage range of -1 to 1 V. This indicates that the BSO thin film exhibits excellent dielectric properties.

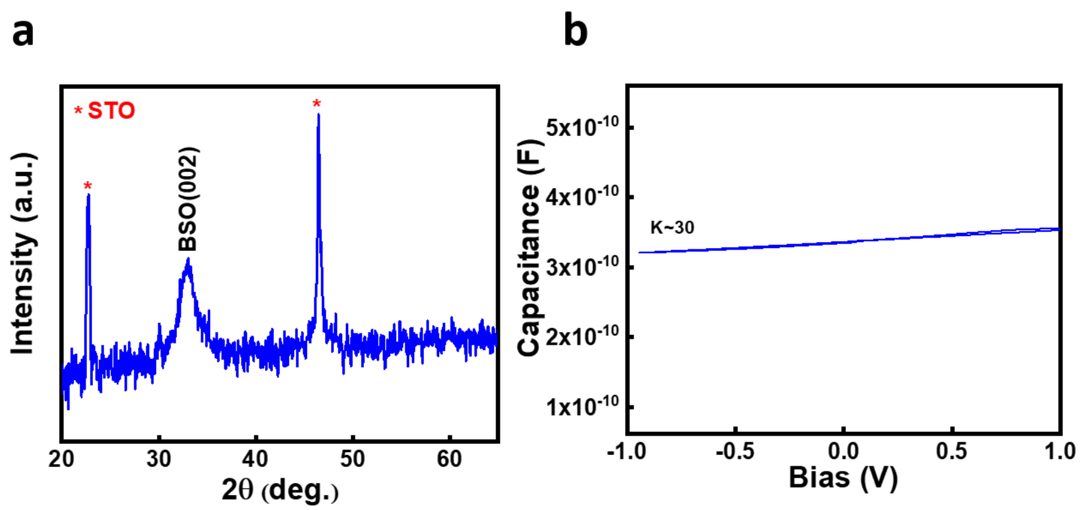

Figure S10. a. The θ-2θ scan result of BSO/STO. b. C-V measurement of BSO thin film.

Device fabrication process flow

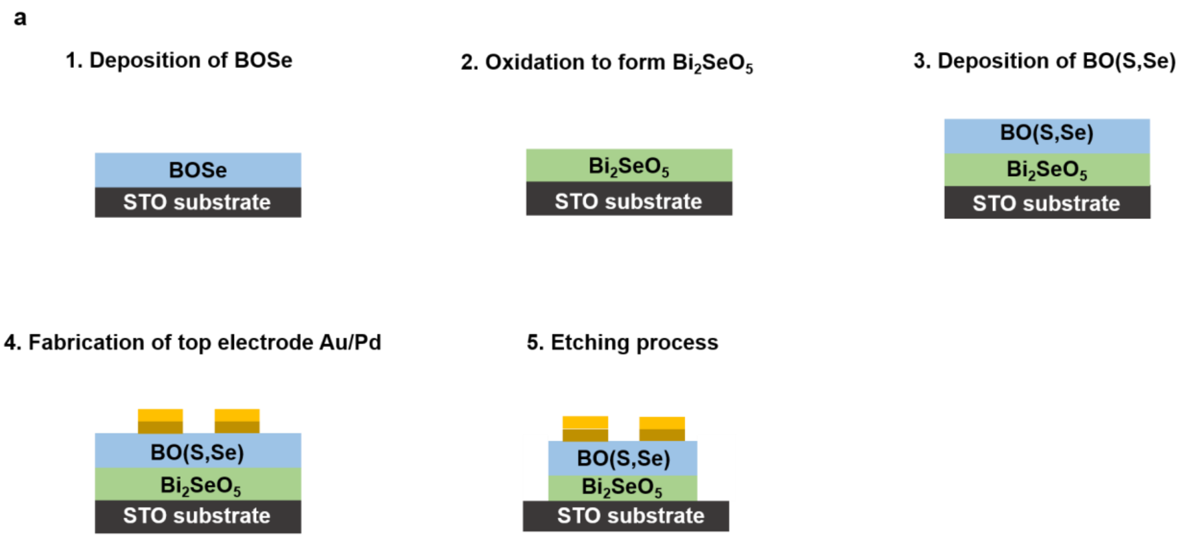

b

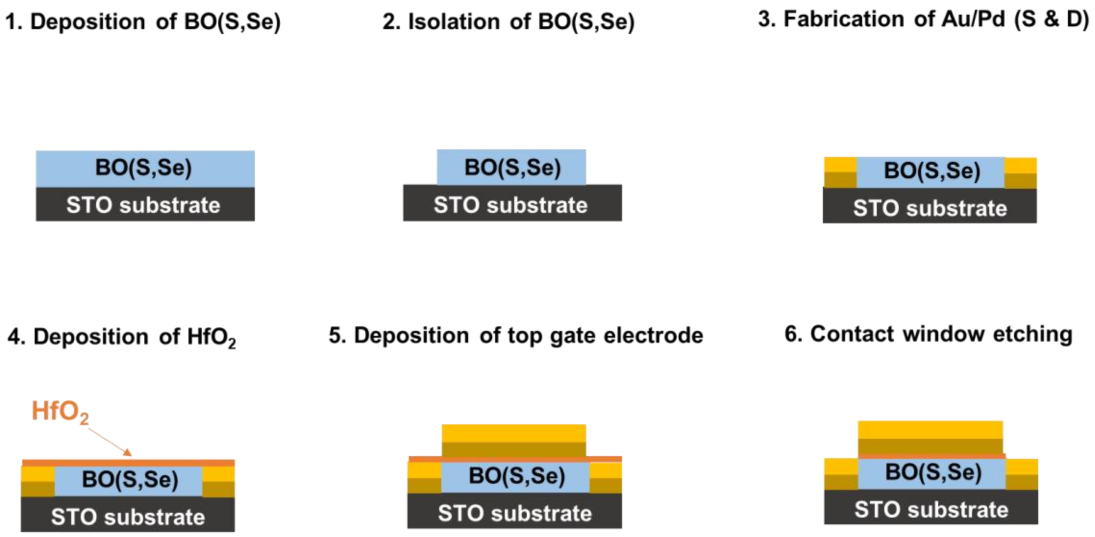

Figure S11. The schematic diagram of a. bottom-gate and b. top-gate device fabrication process flow.
